# The Role of Human Movement in the Transmission of Vector-Borne Pathogens

**DOI:** 10.1371/journal.pntd.0000481

**Published:** 2009-07-21

**Authors:** Steven T. Stoddard, Amy C. Morrison, Gonzalo M. Vazquez-Prokopec, Valerie Paz Soldan, Tadeusz J. Kochel, Uriel Kitron, John P. Elder, Thomas W. Scott

**Affiliations:** 1 Entomology, University of California, Davis, California, United States of America; 2 Department of Environmental Studies, Emory University, Atlanta, Georgia, United States of America; 3 Tulane University, New Orleans, Louisiana, United States of America; 4 United States Naval Medical Research Center Detachment, Lima and Iquitos, Peru; 5 Graduate School of Public Health, San Diego State University, San Diego, California, United States of America; Mahidol University, Thailand

## Abstract

**Background:**

Human movement is a key behavioral factor in many vector-borne disease systems because it influences exposure to vectors and thus the transmission of pathogens. Human movement transcends spatial and temporal scales with different influences on disease dynamics. Here we develop a conceptual model to evaluate the importance of variation in exposure due to individual human movements for pathogen transmission, focusing on mosquito-borne dengue virus.

**Methodology and Principal Findings:**

We develop a model showing that the relevance of human movement at a particular scale depends on vector behavior. Focusing on the day-biting *Aedes aegypti*, we illustrate how vector biting behavior combined with fine-scale movements of individual humans engaged in their regular daily routine can influence transmission. Using a simple example, we estimate a transmission rate (*R_0_*) of 1.3 when exposure is assumed to occur only in the home versus 3.75 when exposure at multiple locations—e.g., market, friend's—due to movement is considered. Movement also influences for which sites and individuals risk is greatest. For the example considered, intriguingly, our model predicts little correspondence between vector abundance in a site and estimated *R_0_* for that site when movement is considered. This illustrates the importance of human movement for understanding and predicting the dynamics of a disease like dengue. To encourage investigation of human movement and disease, we review methods currently available to study human movement and, based on our experience studying dengue in Peru, discuss several important questions to address when designing a study.

**Conclusions/Significance:**

Human movement is a critical, understudied behavioral component underlying the transmission dynamics of many vector-borne pathogens. Understanding movement will facilitate identification of key individuals and sites in the transmission of pathogens such as dengue, which then may provide targets for surveillance, intervention, and improved disease prevention.

## Author Summary

Vector-borne diseases constitute a largely neglected and enormous burden on public health in many resource-challenged environments, demanding efficient control strategies that could be developed through improved understanding of pathogen transmission. Human movement—which determines exposure to vectors—is a key behavioral component of vector-borne disease epidemiology that is poorly understood. We develop a conceptual framework to organize past studies by the scale of movement and then examine movements at fine-scale—i.e., people going through their regular, daily routine—that determine exposure to insect vectors for their role in the dynamics of pathogen transmission. We develop a model to quantify risk of vector contact across locations people visit, with emphasis on mosquito-borne dengue virus in the Amazonian city of Iquitos, Peru. An example scenario illustrates how movement generates variation in exposure risk across individuals, how transmission rates within sites can be increased, and that risk within sites is not solely determined by vector density, as is commonly assumed. Our analysis illustrates the importance of human movement for pathogen transmission, yet little is known—especially for populations most at risk to vector-borne diseases (e.g., dengue, leishmaniasis, etc.). We outline several important considerations for designing epidemiological studies to encourage investigation of individual human movement, based on experience studying dengue.

## Introduction

For vector-borne pathogens heterogeneity in patterns of contact between susceptible hosts and infectious agents is common [1],[2],[3]. Some hosts will be exposed to, harbor, and pass on more parasites than others. Variation in contact patterns can amplify [4],[5] or dampen [6] the rate of transmission, even as it also potentially reduces disease prevalence and epidemic stability (i.e., likelihood of an outbreak; [7]). Understanding and describing what drives heterogeneous contact patterns is thus important for designing improved disease surveillance and prevention programs [3]. If the characteristics of hosts most often infectious or important for transmission are known they could be targeted to more efficiently prevent disease [8]. To be useful for targeted control across different contexts the mechanisms underlying heterogeneous contact patterns must be elucidated. Here we examine the role of individual human movement as a critical behavioral factor underlying observed patterns of vector-borne pathogen transmission, because movement determines exposure to infectious agents; i.e., bites from infected mosquito vectors.

Little is known about individual human movement patterns and even less about their epidemiological consequences, even though such knowledge would be a valuable contribution to the understanding and control of many vector-borne diseases. We begin our investigation of this topic by reviewing studies of human movement. Next, based on an existing typology, we examine the relevance of movement patterns to the dynamics of different diseases. Using the mosquito-borne virus dengue as an example, we develop a conceptual model that illustrates how human and vector behavior can influence pathogen transmission dynamics. We end by outlining key issues important to the design of future research and explaining potential benefits to disease prevention of an improved understanding of host movement.

### A Framework: Movement and Scale

**Figure 1:**
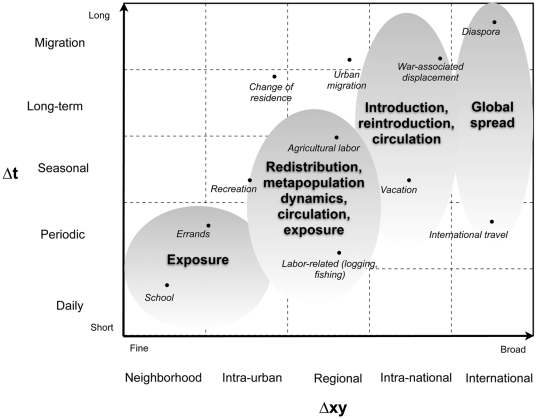
A framework for human movements and their relevance to vector-borne pathogen transmission. Movements are characterized in terms of their spatial and temporal scale, which are defined in terms of physical displacement (*Δxy*) and time spent (*Δt*, frequency and duration). Generally, movements of greater spatial displacement involve more time, but this is not necessarily always the case.

Historically epidemiologists have viewed human movement from the perspective of populations of susceptible hosts moving into high risk areas or infected hosts moving into susceptible populations as explanation for disease occurrence and spread. Indeed, across different scales and diseases, movements of hosts affect pathogen transmission in a variety of ways. Thirty years ago Prothero [9] provided a typology to facilitate study of the role of human movements in epidemiology based on his experience in Africa. Drawing on geography literature concerned with understanding human movement [10],[11],[12], Prothero highlighted the difference between circulatory movements, where individuals return home after some period, and migratory movements, which tend to be permanent changes of residence (see Figure 1 in [11]). He further characterized movements by their ‘spatial scale’, which he categorized in terms of a rural-urban gradient, and temporal scale based on the time and timing of displacements. He qualified these categories in terms of their relevance to public health. For instance, seasonal movements from one rural area to another for agriculture could potentially expose individuals to different ‘ecological zones’ where the risk of malaria or African trypanosomiasis is high [13]. His argument was that knowing something about the nature of such movements would help explain the incidence and prevalence of disease in a population and provide informed options for control [9]. In Figure 1 we generalize Prothero's typology in terms of the spatial and temporal scale (*sensu* [14]) of human movement and extend it to include most vector-borne disease contexts.

At broad spatial scales (e.g., national, international) individual movements drive pathogen introduction and reintroduction (far right, Figure 1). Global spread of dengue virus via shipping routes was characterized by periodic, large, spatial displacements [15]. Globalization and air transportation have changed the dynamic of pathogen spread by dramatically shortening the time required to travel around the globe [16],[17],[18]. The recent chikungunya epidemic in the Indian Ocean that subsequently spread to Italy is an example [19]. At finer scales (e.g., regional, urban-rural, intra-urban; far left of Figure 1), movement associated with work, recreation, transient migration, and other phenomena is important to patterns of pathogen transmission and spread [9],[20]. Movements into high-risk areas not only lead to individual infection, but can also contribute to local transmission when infected hosts return home and infect competent vectors. For example, in the Chocó region of Colombia most malaria transmission occurs in rural areas and many cases diagnosed in the city of Quibdó are due to travel to these areas [21]. Transmission also occurs locally within Quibdó [22], however, most likely because of infected travelers returning and infecting competent vectors. Understanding the origin of infections and the relative importance of human movement at different scales to both local and regional transmission dynamics would increase effectiveness of disease prevention programs by, for example, identifying individuals at greatest risk of contracting and transmitting pathogen.

Generally, a key significance of human movement for vector-borne disease at any scale lies with exposure to vectors. Exposure is local in space and time and variation in exposure due to individual host movement could strongly influence the transmission dynamics of pathogens. For instance, circulatory movements associated with working in rural areas and variation in movement patterns among cultures may explain heterogeneous patterns of onchocerciasis incidence. While men in Cameroon and Guatemala both experience similar parasite loads reflecting exposure to vectors when working in fields, women in the 2 countries show different patterns of infection partly due to differences in exposure [23]. The type of movement most relevant for exposure will depend on site specific differences, the ecology of the arthropod vector, human behavior, and the relative scale of host and vector movement. For pathogens transmitted by vectors able to move long distances in search of a host, fine scale host movements may not be important, while they are for pathogens transmitted by sessile vectors. *Aedes aegypti*—the principal vector of dengue virus—bites during the day [24], disperses only short distances [25] and is heterogeneously distributed within urban areas [26],[27]. Conversely, humans move frequently at local scales (bottom-left of Figure 1), allocating different amounts of time to multiple locations on a regular basis. This will influence individual risk of infection with dengue virus [28] and thus overall patterns of transmission [29],[30],[31].

## Methods

The dynamics of human movement, the locations used and the paths between them, is conceptualized by the ‘activity space’ model developed in the 60's by human geographers [12],[32],[33]. This model, much like the ‘home-range’ concept in ecology, is effective because organisms exhibit habitual behavior in their use of space [34]. For our purposes of studying dengue, the ‘activity space’ refers to those few locations where humans commonly spend most of their time [32],[35] and ‘movement’ refers to the use of these locations. Thus, exposure to host-seeking female *Ae. aegypti* is the sum of exposure across an individual's activity space. For other vectors and pathogens, human movements *per se* (e.g., walking between the house and a water source) and/or visits to less common destinations could be relevant for the transmission of other pathogens (e.g., African trypanosomiasis) depending on the behavior of the vector and the relative scales of vector and host movement.

**Figure 2:**
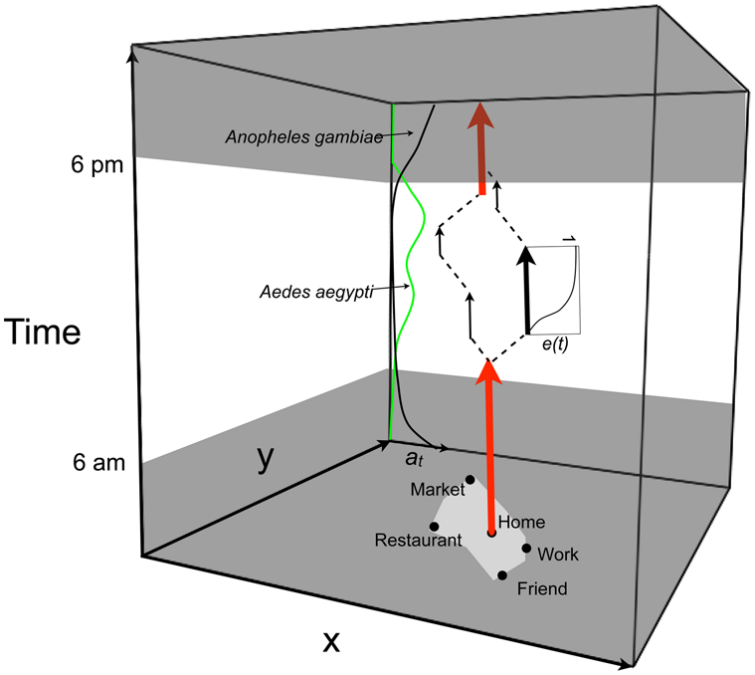
The activity space model. Space is plotted in the xy plane and time on the z axis. In this example daily movements for a week are represented. Points in the xy plane are sites visited and the polygon depicts the activity area. Vertical arrows indicate time spent at a site. Thickness of arrows indicates frequency of visitation and length shows duration. Red arrows are for the home and here we assume a person is in the home every night of the week. Dashed lines represent movement between sites with velocity indicated by the angle of the line. Grayed-out regions of the cube represent night-time. Not shown is variation in vector abundance among sites. Plotted along the back axis for time are representative curves of biting rates, *a(t)*, for *Ae. aegypti* (green), a day biting mosquito, and *Anopheles gambiae* (black), a night biting mosquito. Plotted to the right of the large black arrow is a cumulative biting probability, *e(t)*, as a function of time spent in the location. See text for more detail.

The activity space model represents movement associated with the regular activity of individuals [36]. We present a version of this model in Figure 2 for understanding how movements within an urban area might contribute to risk of exposure. Risk at locations within an individual person's activity space will vary depending on the number of infected, host seeking vectors present and their biting behavior. For instance, visits to locations during the day are of minimal risk for bites from nocturnal *An. gambiae*, but are relatively high for day active *Ae. aegypti* (Figure 2). Exposure to vector bites may also depend on how long a person stays at a given location. If vectors are stimulated by the arrival of an individual to a location (as may be the case for *Ae. aegypti* and *Aedes albopictus* [37],[38]), then a bite is most likely to occur early after arrival (i.e. the cumulative probability of a bite during a visit, *e(t)*, accumulates rapidly). Alternatively, for vectors like triatomine bugs, which are less opportunistic than mosquitoes, long visits will be expected to pose a higher risk of host-vector contact (*e(t)* slowly accumulates over time). How vectors respond to hosts arriving at a site is important because it weights the risk of visits differently depending on their frequency and duration. If a vector is stimulated to host seek by the arrival of a host, then multiple short visits to that site will carry greater risk than a single long visit of equivalent total duration.

In summary, a person's risk of exposure to an infective vector can be represented with a simple exposure model for indirectly transmitted disease:
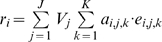
(1)


Here, the risk of exposure (i.e., being bitten by a vector) for individual *i*, *r_i_*, over some observation period is simply the sum across sites visited, *j*, of vector abundance, *V_j_*, conditioned on the time and duration of all visits to that site, *k*, as determined by vector behavior (where *K* is the total number of visits during the observation period). The biting rate, *a_k_*, is the number of bites expected per visit and is drawn from the day biting rate distribution for the times of the visit.
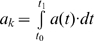
(2)


How vectors respond to the appearance of a host at a site is captured by *e_k_*, the cumulative probability of a bite given the time spent in the site, and is bounded by the unit interval.
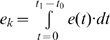
(3)


Visits, *k*, are defined by an arrival time, *t_0_*, and a departure time, *t_1_*, in hours and are in reference to a single day. At the limit (where *t_1_*−*t_0_* = 24 hours), *a_k_* becomes the day biting rate, *a*, and *e_k_* goes to 1 and we recover the model often assumed for vector-borne diseases where exposure occurs in the household. Note that although we imply here that a site comprises a household or other edifice because of our focus on dengue, in truth it simply demarcates a location where the abundance and activity of vectors is independent of other locations and is defined by the scale of vector movement.

Site-specific exposure risk is calculated as:
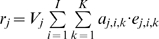
(4)and has units of bites*humans for the observation period. Note that in this formulation, risk among individuals using the same site is assumed to be independent (i.e., the expected number of bites at a site is the product of humans present and vector activity). This may not be realistic if hosts occupy a site at the same time, which would be expected to dilute the number of bites individual hosts receive, and can be corrected (see below) by incorporating the actual amount of time individual humans spend in a location. The estimate of risk, *r_j_*, can be used to estimate the transmission rate, *R_0_*, which is the number of secondary infections expected from the introduction of a single infective individual into a wholly susceptible population. Woolhouse et al. (1997) use the following approximation for *R_0_*:
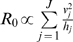
(5)where *v_j_* is the proportion of vectors at site *j*, *h_j_* is the proportion of hosts living in site *j*, and *J* is the total number of sites. Risk as estimated above is incorporated by replacing *v_j_* with site associated risk, *r_j_*, discounted by the proportional use of that site within some interval by people, *h_j_*:
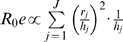
(6)


For example, if a site is used by 2 individuals for 6 hours each over a week, *h_j_* = (2 humans * 6 hours)/(24 hours/day * 7 days) = 0.07 humans. The activity space model elaborated here illustrates that host and vector behavior are very important for determining who gets bitten and has the greatest risk of contracting or transmitting a pathogen.

## Results

**Figure 3:**
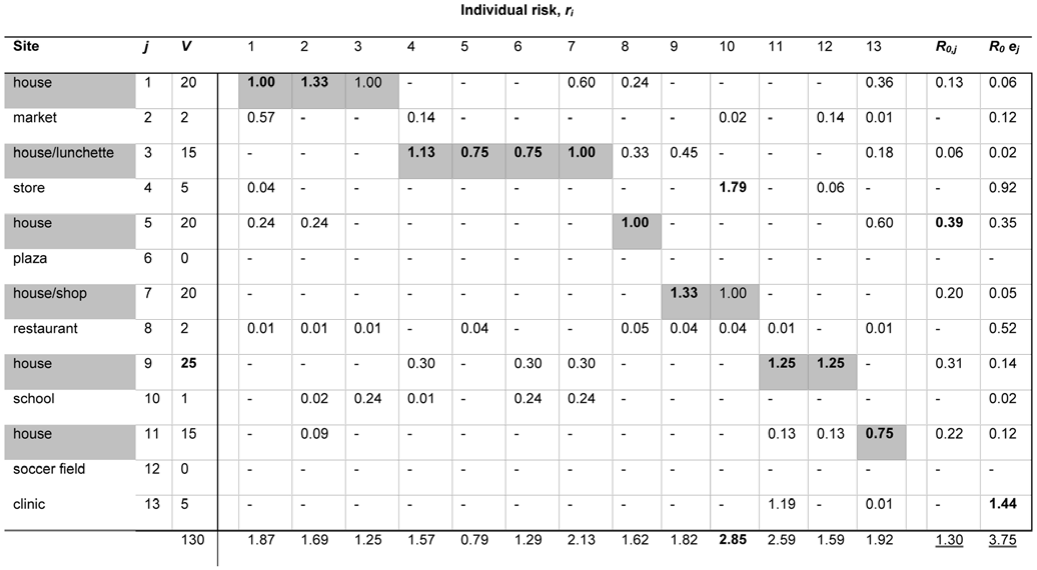
Example scenario of risk of exposure due to individual movements. Individuals (*i*, represented by columns) live in and visit a number of sites (*j*, rows) for different durations and frequencies during a regular week. Each site is infested with a number of female mosquitoes, *V*. Grey shading indicates the home of each individual. Risk of a mosquito bite, *r_i_*, is calculated as described in the text and is presented here for each individual given the number of visits and time spent at different locations during a typical week. Numbers in bold are maxima for each column. Here the probability of a mosquito bite at night (in the home) is assumed to be 10% of all other times. The sum of individual risk is shown along the bottom of the figure. Overall transmission rate estimated without, *R_0_*, and with exposure, *R_0_ e*, considered are shown in the bottom-right and underlined. See Text S1 and Table S1 for further details.

The activity space model when coupled with our knowledge of vector behavior provides a tool for determining what human movements are important for transmission (e.g., Figure 1). Specifically, it allows us to identify places and individuals that contribute disproportionately to pathogen transmission dynamics. For example, consider the following scenario depicted in Figure 3 for a human population at risk for dengue virus infection like the one we are studying in Iquitos, Peru (Figure 3, Text S1 and Table S1). Briefly, individuals spend their time at a number of different sites, both commercial and residential, during their regular weekly activities (Sites, first column in Figure 3). Sites have different numbers of female mosquitoes and are visited at different rates and for different durations. We can estimate the risk of exposure to host-seeking female mosquitoes (*r_i_*) for each person (columns 1–13 in Figure 3) at each site (rows in Figure 3) and then estimate *R_0_*. In this particular example, *R_0_* as approximated when accounting only for the home (eq. 5) is 1.3 and the site with the highest estimated risk is house 5 (in bold in column under *R_0_*). If we account for exposure at all locations in addition to the home and assume the biting rate at night is 10% of the rate during the day [39], our estimate of *R_0_* (eq. 6) jumps nearly 3-fold and the most important site is 13, a clinic (in bold under *R_0_e*). This latter result arises because of the relatively large number of bites per person expected at that site, determined largely by the significant amount of time a single person spends there (e.g., their workplace). In this example, all individuals except individual 10 experience the greatest exposure to bites in their homes because that is where they spend the most time. Individual 10, however, experiences the highest risk at site 4, which represents their workplace. This individual is also at the greatest risk in the host population.

**Figure 4:**
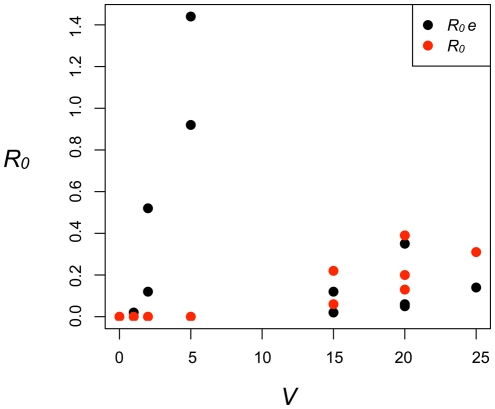
Estimates of *R_0_* plotted against vector density at sites. *R_0_* is calculated assuming exposure occurs only within homes, *R_0_ e* is calculated taking exposure rates into account based on representative activity patterns of several hypothetical individuals living in a community like Iquitos, Peru, where we are studying dengue transmission (Figure 3).

This example illustrates that the key sites are not necessarily those of greatest vector abundance, as is commonly assumed. For this example scenario, *R_0j_* increases monotonically with vector abundance when transmission is assumed to occur only in the home (Figure 4). When exposure rates are accounted for, however, there is no relationship between *R_0j_* and vector abundance (Figure 4). Similarly, people living where vector abundance is greatest are not necessarily at greater risk. Human movement and subsequent variation in exposure thus becomes more important than vector density *per se*. Because heterogeneity in contact patterns has a large influence of the rate of pathogen transmission, variation in exposure rates due to individual movement patterns could have considerable impact on disease dynamics [40],[41].

## Discussion

To fully understand the implications of movements, however, data should be incorporated into network, individual-based or metapopulation models [5],[42],[43]. Network models, in particular, capture heterogeneity explicitly and intuitively, allowing precise prediction of trends and patterns in human infection and disease [44]. For dengue, one imagines a dynamic network of individuals most likely to become infected or infect mosquitoes and of locations where transmission is most likely to occur [29]. These are the key nodes of pathogen transmission that, if identified and understood, would be excellent targets for intervention (e.g., [8]).

The value of estimating actual exposure rates and incorporating these into models to better understand pathogen dynamics is clear for dengue, which is mostly transmitted when people are engaged in daily activities [29]. For this reason we are currently monitoring human movements in Iquitos, Peru. The activity space model as we describe it, however, highlights that movements may be important for the transmission of many pathogens typically thought to be transmitted at night when hosts are inactive. Sand fly vectors of American visceral leishmaniasis are active at dusk [45], move short distances [46], and are heterogeneously distributed among homes [47], which, in combination with human behavior, may be key to understanding leishmaniasis incidence patterns [48],[49]. Similarly, Michael et al. [50] found that 27% of *Culex quinquefasciatus* resting within households had fed on hosts from outside that home despite its nocturnal habit, with implications for transmission of lymphatic filariasis. There are thus many reasons for increased examination of individual human movement patterns.

### Measuring Movements

As an aid to future research, in the remainder of this article we discuss key issues and considerations for designing studies of human movement based on our experiences with dengue.

#### Spatial scale

The first question to ask when one seeks to measure human movements and evaluate their role in pathogen transmission concerns spatial scale. This can be determined by the disease dynamic of interest; e.g., spread of a pathogen to new geographic areas vs. sustained transmission at a given locale. If the question concerns local transmission, then relevant movements will be those placing susceptible hosts in high risk locations at times when infection risk is high. General information about a particular system may guide this process. Assumptions regarding the importance of movements should be made cautiously because heterogeneity in exposure can have a dramatic effect on infection risk.

#### Type of movement

Next, one should ask what to measure. The term ‘movement’ is used somewhat ambiguously. Are we interested in just the sites where individuals spend their time on a regular basis (high spatial and temporal resolution) or whether they are in the home/city or elsewhere? Do we want travel information (outside of an urban area) that specifies exactly where people go or just a general notion? Are specific routes important, or should only destinations be considered? These details will, again, depend on the question, system, and resources and methods for measuring movements.

Where we work in Peru, dengue transmission is primarily focused in urban areas of Iquitos and the mosquito vector, *Ae. aegypti*, is not found in the majority of rural areas outside of the city. As such, we are comfortable excluding movements to rural areas because people are unlikely to be infected there. We only need know that they were not in Iquitos, and where they were is only important if that location has dengue as well. If we were studying malaria, we might do the opposite and ignore movements within urban Iquitos where malaria is not transmitted. In our study of local dengue transmission we want high spatial and temporal resolution because *Ae. aegypti* cluster at the scale of individual households and bite during the day [26]. For malaria, regional movements to and from fishing or logging camps are a likely dynamic driving transmission patterns and simply knowing to which camps individuals move to on a periodic or seasonal basis and the routes taken should be sufficient to understand the spatial dynamics of that disease (G. Devine, personal communication).

#### Measurement method

A third question concerns how to measure movements. A number of methods and technologies are currently available that allow tracking of individual movements (Table 1). The choice of the appropriate method is dependent on the scale of the study and the disease in question. If the scale of interest is broad, then data from transit networks may be suitable, as has been done in studies of the global spread of SARS and influenza [17],[51],[52]. For finer scales, lack of appropriate means for measuring movements is one reason so little has yet been done in a rigorous, quantitative fashion (Vasquez-Prokopec et al. unpublished). The technology has long been available in some form, but has proved too cumbersome and expensive for large scale use with humans. Indirect devices commonly used in the social sciences, such as travel diaries, are a good source of information when used rigorously, but have seen limited use in the study of indirectly transmitted pathogens, perhaps because of inherent bias and imperfect recall.

**Table 1:** Methods for measuring human movement.

Method	Description	Pros	Cons	Ideal use
Recall	Commonly used in studies of exercise and physical activity, in diary or close-ended formats	Captures both quantitative and qualitative information; used internationally in chronic disease research.	Subject to memory decay, social desirability, and other response biases. Have been used primarily in developed countries.	Not as primary outcome but to validate and inform electronic instrumentation and other more objective measures
Telemetry	Commonly used in wildlife studies, involves a transmitter placed on an individual and antennas (fixed or mobile) for locating the transmitter.	Can be inexpensive, long battery life of transmitters, well established method, range dependent.	Short range, Difficult to get precise location information, expensive for large scale use (i.e. establishing an array of antennas), interference in urban areas.	Wildlife diseases, not practical for use with humans.
RFID	Radio Frequency Identification Device, used to track inventories, individuals in hospitals. Involve a small ‘tag’ and an antenna to detect tag.	Tag is very small, easy to wear, and battery lasts a very long time.	Short range, requires network of antennas to track movements in an area, which can become expensive.	Very good option for tracking movements to and from predefined locations, e.g., for movements to commonly used water sources.
GPS	Global Positioning System. Global, satellite-based, location aware system.	Only requires a receiver, works everywhere, provides exact positional information, devices are becoming very small and inexpensive.	Large data post-processing requirement, short battery life, custom devices are expensive while commercial options not tailored to research use.	Reductions in cost and device size make GPS the best option for tracking movements where cellular phone use is not universal.
GSM-GPS	GSM assisted GPS. Devices use the GSM cellular network to improve the satellite signal and provide positional information when satellites are out of reach due to interference.	Same as GPS with the additional benefit of location information inside buildings and other places the satellite signal cannot reach.	Additional positional information depends on cellular network, feature requires data transmission, network fees and arrangements necessary, very short battery life.	Because the additional advantage of these devices relies on a cellular network, either GPS or cellular phones will often be better options.
Cellular phone	The position of cellular phones can be approximated through triangulation using the cellular network.	Where cellular phone use is universal, movement data can acquired from network providers without any inconvenience to study participants.	Potential for bias (positions are recorded when phones are used), low spatial precision, requires network agreement, privacy issues, most individuals need personal phones.	For large scale studies of the collective dynamics of populations, regional movements and movements within large metropolitan areas
Cellular phone, AGPS	Assisted-GPS on cellular phones works by the same mechanism as GSM-GPS, utilizing the cellular network to assist in acquiring positional information.	High spatial precision, potential for high coverage where cellular phone use is common, no need to purchase devices.	Dependent on cellular network, requires data transmission, may require custom software or other means to acquire data while avoiding privacy issues. Can be very expensive without a special arrangement with a network provider.	Most useful for studying movements in developed countries were cellular network coverage is high and most people have personal phones. Also good for urban areas where GPS signal is imperfect.

All available technologies have pros and cons (Table 1). GPS has often been considered to measure exposure, but because of cost, size, battery life, and other technical limitations has yet to be used incisively to study human movements (Vasquez-Prokopec et al. unpublished). Cellular phones hold promise where the technology is available and use is universal (e.g., [35]), but are awkward to use for prospective studies and in low-resource settings and come with privacy issues. GPS seems to hold the greatest potential for the combination of low cost, ease of use, spatial accuracy, and fewer privacy issues than cellular phones because only location information is recorded. We are currently using a GPS device in Iquitos, Peru that weighs less than 25 g, records for >3 days continuously, and is under $50 (Vasquez-Prokopec et al. unpublished). Size and battery life of tracking devices are critical in human studies because they are key to acceptance by participants for long term use (minimizing coverage bias, Paz Soldan et al., unpublished, [53]). Except for cellular phones owned by participants, any currently available device is only useful for prospective investigation.

To evaluate the role of movements on disease dynamics retrospectively–that is, after identifying an infected individual–the options are limited. Cellular phones may be useful in certain contexts: e.g., where the technology is accepted and widely used (Table 1). Otherwise, instruments reliant on recall such as diaries, questionnaires, or interviews are required. These methods are imperfect, yet can provide valuable information when coupled with other tools. For instance, Geographic Information Systems permit production of detailed maps for a region that can be used to elicit recall of visits to certain sites (Paz Soldan et al. unpublished). Recall instruments should be sensitive to the local social and cultural contexts. As such, active collaboration with social scientists versed in the local culture is critical for the development of an interview device with sufficient sensitivity. Technologies such as GPS can be used to facilitate development of a recall instrument and to validate it. Location aware technologies, however, are not a gold standard for measuring movement because of precision and accuracy limitations, problems of compliance and use (Paz Soldan et al. unpublished), and other factors that can disrupt tracking (e.g., interference from buildings). Moreover, GPS does not indicate an individual's activity, which could be critical for determining risk [e.g., did they enter the house or stay on the sidewalk? 53]. Combining objective (e.g., GPS) and recall methods may be the best way to efficiently follow individual movements on a large scale and to qualify those movements with regard to disease risk.

#### Observation interval

A fourth question concerns how long to observe individual movements. The answer will depend on the question being asked and available resources. In the case of dengue, infection can occur up to 2 weeks prior to the manifestation of symptoms. For a retrospective study, 14–15 days would be the right observation period. Conversely, in a prospective study the length of the observation period will depend on the relative importance of rare movements. Studies of human movements in developed societies reveal markedly regular patterns, especially during the work-week [32],[35],[54],[55],[56]. Conversely, there may be significant instability in movements on weekends or at other times (e.g., vacations). For regular movements during the work week, at least 2 weeks of observation are needed. For more variable movements/times, substantially longer observation periods will be necessary [54]. The need for long-term observation reinforces the need to ensure acceptability of tracking devices by the study population and emphasizes the importance of device wearability (Paz Soldan et al. unpublished, Vasquez-Prokopec et al. unpublished).

#### Data management

Although gathering movement information is becoming more feasible, handling movement data remains a challenge [53]. GPS and other devices provide tracks of movements that must be processed into data useable in analyses: for example, the locations visited, frequency of visitation, and time spent during visits. Tools are becoming available to facilitate data processing (e.g., [57],[58],[59]) that integrate with existing GIS and statistical software packages (e.g., Arc-GIS, R). Such tools will facilitate data analysis.

### Conclusions

Because patterns of contact between pathogens and susceptible hosts are heterogeneous, disease interventions can be made more effective and efficient by targeting the key points or ‘nodes’ of transmission [3]. Even where heterogeneous patterns are clearly documented, not knowing the factors driving such patterns impedes one's ability to effectively target control. Is a biting preference toward young adults [60] because they are intrinsically more attractive to a host-seeking mosquito or, because of their behavior, they are more likely to be exposed to mosquitoes? Although many different causes of host-vector contact heterogeneity have been proposed [summarized by 6], variation in exposure due to human behavior is likely to be key across disease systems. The role of other risk factors (e.g., host-preference) will always be conditioned by exposure rates. The study of human movement is thus critical to the identification of key individuals and key locations. Nevertheless, movements have largely been neglected in studies of indirectly transmitted disease even though it is becoming increasingly easy to measure.

Quantifying and describing human movements promises more than just characterization of key heterogeneities. Quantification of the collective dynamics of human populations provides information necessary for models intended to predict disease outbreak and spread and to evaluate control alternatives to halt epidemics [8],[35],[51]. Buscarino et al. [61], for instance, predict that movements within a population have an important effect on the epidemic threshold, lowering this as individuals move over larger distances more frequently. Additionally, quantifying movements and applying that information to a variety of diseases creates the opportunity to identify common places where infection occurs across diseases and, thus, the potential to leverage public health programs by allowing limited resources to be targeted to the most important locations for more than one disease.

Rigorous examination of the role of human movement across different scales will significantly improve understanding of pathogen transmission, which will be critical to increasing the effectiveness of disease prevention programs. As transmission rates are reduced through intervention efforts, we expect the importance of heterogeneity in exposure to increase and to play an even more important role in pathogen persistence. Characterization of movements will thus not only facilitate the elimination of disease, it will help to prevent its return.

## Supporting Information

Text S1 Calculating individual risk.
(0.06 MB DOC)

Table S1 Example space time budget from human movements.
(0.05 MB XLS)
