# Supplementary material for: The Role of Human Movement in the Transmission of Vector-Borne Pathogens
Source: PLoS Negl Trop Dis. 2009 Jul 21;3(7):e481. doi: 10.1371/journal.pntd.0000481 (PMC2710008; doi:10.1371/journal.pntd.0000481)
Supplement: Text S1 — Calculating individual risk. (0.06 MB DOC) [file pntd.0000481.s001.doc]

**Supplemental information**

Using the equations presented in the main text, one can calculate individual and site exposure risk by quantifying a space-time budget for individuals using GPS, interviews, or other means. An example is in Figure S1, (see also Table S1). In Figure S1 a single individual allocates their time among a number of different sites. The time spent at each location each time they visit, *Δt,* is in minutes. The number of times a site is visited in a period of time (e.g., a week) is given under *f*. For simplicity, we assume here that each visit to a site is at the same time of day and for the same length of time. The probability of getting bitten by a mosquito as a function of the time of day the person is in a site is given by *a(t).* For this simple example, we treat *a(t)* as a probability of a bite as opposed to an expected number of bites permitting direct comparison to the approximation of *R_0_* used by Woolhouse et al. 1997, which uses vector density only as opposed to bites to estimate transmission. Here we assume that biting at night is 10% of all other times, based on expected behavior of a day-biting vector, and that individuals are in their home (grey highlight) at night. In this example we also assume that the probability of a mosquito bite given the duration of a visit is constant (i.e., *e(t)* = 1) and that all individuals have the same probability of being bit. Each site is infested with mosquitoes, *V*, which determines the risk of a bite given the time an individual spends at a site.

In this case, the risk of a bite in a site is estimated as *V * (Δt * f )/ (dt * P) * a(t).* Here the time interval, *dt,* = 1 minute and *P* = 10080 minutes*week^-1^.

In Figure S1, the individual spends 12 hours a day, 7 days a week in the home (e.g., 8 pm to 8 am), works at the market 8 hours a day Monday thru Saturday, goes to the store 3 times a week, visits a friend or relative a couple times a week, and goes out to eat once a week. Based on this activity pattern and the number of vectors at the sites visited, the expected number of bites this person would receive is 1.87 per week, most of which would occur in their home and workplace (note the use of ‘bites’ here is misleading, as the estimate is actually of the number of encounters with biting mosquitoes because for this example *a(t)* is treated as a probability as opposed to an expectation of the number of actual bites as would normally be used in estimates of vectorial capacity, for instance).

| Figure S1 | | |  | |  | i1 |  |  |  |
| --- | --- | --- | --- | --- | --- | --- | --- | --- | --- |
|  | | | *j* | | *V* | Δ*t* | *f* | *a(t)* | *ri* |
| house | | | 1 | | 20 | 720 | 7 | 0.1 | 1.00 |
| market | | | 2 | | 2 | 480 | 6 | 1 | 0.57 |
| house/menu | | | 3 | | 15 |  |  | 1 | 0.00 |
| store | | | 4 | | 5 | 30 | 3 | 1 | 0.04 |
| house | | | 5 | | 20 | 60 | 2 | 1 | 0.24 |
| plaza | | | 6 | | 0 |  |  | 1 | 0.00 |
| house/shop | | | 7 | | 20 |  |  | 1 | 0.00 |
| restaurant | | | 8 | | 2 | 60 | 1 | 1 | 0.01 |
| house | | | 9 | | 25 |  |  | 1 | 0.00 |
| school | | | 10 | | 1 |  |  | 1 | 0.00 |
| house | | | 11 | | 15 |  |  | 1 | 0.00 |
| soccer field | | | 12 | | 0 | 60 | 1 | 1 | 0.00 |
| clinic | | | 13 | | 5 |  |  | 1 | 0.00 |
| Total | | |  | | 130 | 8250 |  |  | 1.87 |
|  |  |  | |  | | | | | |

Calculating risk for a number of sites and individuals allows estimation of *R_0_* (see main text). To calculate these estimates using a space-time budget we first sum individual risk for each site and then divide that total by the proportion of time that site is used in the observation period, *h_j_* (e.g., a week). For instance, if 3 individuals in a total population of 13 use site 1 for 12 hours a day, 7 days a week, human time at that site is (3 people * 720 minutes days^-1^ * 7 days week^-1^) / (10080 minutes week^-1^ * 13 people) = 0.115 (see Table S1). Thus, this site is used at a rate of 11.5% of the total possible (i.e. if all the population spent all their time in that site). In this way comparison may be made to the approximation used by Woolhouse et al (see main text) in order to illustrate the importance of exposure at the many sites an individual visits. The Woolhouse et al approximation, in fact, is equivalent to calculation of site risk assuming *a(t)* = 1 and *e(t)* = 1 and all time is spent in the household.

| **Table 1:** Definition of parameters in Table S1 | |
| --- | --- |
| **Parameter** | **Description** |
| *j* | site reference |
| *V* | number of female mosquitoes |
| *Δt* | duration of visits to site *j* |
| *f* | frequency of visits to site *j* within some interval, e.g. 1 week |
| *a(t)* | probability of biting as a function of the time of day of a visit |
| *r_i_* | individual risk: *V * (Δt * f )/ (dt * P) * a(t)* |
| *r_j_* | site risk: *Σ r_i_* |
| *h_j_* | human host time at location *j* |
| *R_0_* | pathogen transmission rate |
| *R_0_ i* | pathogen transmission rate per individual |
| *R_0_ e* | pathogen transmission rate per site with exposure included |
